# M. Trousseau on Urtication in the Treatment of Rubeola

**Published:** 1844-02-10

**Authors:** 


M. TROUSSEAU ON URTICATION IN THE TREATMENT OF RUBEOLA.

In a recent number of the "Journal de Medecine," after recalling the wise precepts of Sydenham, who advises not to use stimulants in the treatment of persons labouring under eruptive fevers, and not to keep them too warm. M. Trousseau remarks that, although these precepts are generally correct, we must not entirely proscribe the employment of local or external excitants when the exanthema has disappeared abnormally. He then states that in several instances he has resorted with great advantage to urtication, and gives the following case:—A young woman was received in his wards on the sixth day, of a rubeola. The eruption was very intense, but on the day following her admission she was seized with very acute capillary bronchitis, and the eruption disappeared. Copious bleeding was resorted to without success, and the dyspnœa became so intense, the pulse was so small and frequent, that he despaired of her life. He then gave a sufficient dose of ipecacuanha to produce vomiting, and when she had recovered from the state of semi-syncope into which the vomiting had thrown her, he had her severely fustigated with nettles from the head to the feet. The effect was truly magical. In the course of a few hours after the remedy had been resorted to, the oppression had ceased, the fever had nearly disappeared, and convalescence had commenced.—*London Lancet.*

